# Alteration of Branched-Chain and Aromatic Amino Acid Profile as a Novel Approach in Studying Polycystic Ovary Syndrome Pathogenesis

**DOI:** 10.3390/nu15194153

**Published:** 2023-09-26

**Authors:** Katarzyna Paczkowska, Dominik Rachoń, Andrzej Berg, Jacek Rybka, Katarzyna Kapczyńska, Marek Bolanowski, Jacek Daroszewski

**Affiliations:** 1 Department of Endocrinology, Diabetes and Isotope Therapy, Wroclaw Medical University, 50-367 Wroclaw, Poland; katarzyna.paczkowska@student.umw.edu.pl (K.P.);; 2 Department of Clinical and Experimental Endocrinology, Medical University of Gdansk, 80-211 Gdansk, Poland; 3 Department of Pharmaceutical Chemistry, Medical University of Gdansk, 80-416 Gdansk, Poland; 4 Laboratory of Medical Microbiology, Hirszfeld Institute of Immunology and Experimental Therapy, Polish Academy of Sciences, 53-114 Wroclaw, Poland

**Keywords:** PCOS, amino acid profile, branched-chain amino acids, aromatic amino acids, metabolically healthy obesity

## Abstract

Polycystic ovary syndrome (PCOS) is a common endocrine disorder that affects reproductive-age women and predisposes them to the development of metabolic disturbances. Recent research has shown that several metabolic factors may play a role in PCOS pathogenesis, and it has been suggested that an alteration in the amino acid profile might be a predictive sign of metabolic disorders. Metabolically healthy obesity (MHO) and metabolically unhealthy obesity (MUO) are concepts that have attracted scientific attention; however, a universal definition has not been established yet for these terms. Already existing definitions of MHO involve the coexistence of obesity with the absence or minimal presence of other metabolic syndrome parameters. A group of 326 women, 209 diagnosed with PCOS and 117 healthy individuals, participated in this study. Multiple parameters were assessed, including anthropometrical, biochemical, and hormonal ones, and gas–liquid chromatography, combined with tandem mass spectrometry, was used to investigate the amino acid profile. Statistical analysis revealed noticeably higher levels of all aromatic amino acids in PCOS women compared to the control group: phenylalanine 47.37 ± 7.0 vs. 45.4 ± 6.09 nmol/mL (*p* = 0.01), tyrosine 61.69 ± 9.56 vs. 58.08 ± 8.89 nmol/mL (*p* < 0.01), and tryptophan 53.66 ± 11.42 vs. 49.81 ± 11.18 nmol/mL (*p* < 0.01); however, there was no significant difference in the “tryptophan ratio” between the PCOS and control group (*p* = 0.88). A comparison of MHO and MUO PCOS women revealed that LAP, leucine, and isoleucine concentrations were significantly higher among the MUO subgroup: respectively, 101.98 ± 34.74 vs. 55.80 ± 24.33 (*p* < 0.001); 153.26 ± 22.26 vs. 137.25 ± 25.76 nmol/mL (*p* = 0.04); and 92.92 ± 16.09 vs. 82.60 ± 18.70 nmol/mL (*p* = 0.02). No significant differences in BMI, fasting glucose, and HOMA-IR between MHO and MUO were found: respectively, 35.0 ± 4.8 vs. 36.1 ± 4.6 kg/m^2^ (*p* = 0.59); 88.0 ± 6.0 vs. 87.73 ± 6.28 mg/dL (*p* = 0.67); and 3.36 ± 1.70 vs. 4.17 ± 1.77 (*p* = 0.1). The identification of altered amino acid profiles in PCOS holds potential clinical implications. Amino acids may serve as biomarkers for diagnosing and monitoring the metabolic status of individuals with PCOS. The alteration of BCAAs and AAAs may be involved in PCOS pathogenesis, but the underlying mechanism should be further investigated.

## 1. Introduction

Polycystic ovary syndrome (PCOS) is a complex endocrine disorder characterized by hyperandrogenism, anovulation, and a polycystic morphology of the ovaries. It is a heterogenous disorder with different clinical presentations; a diversity in phenotypes, such as the presence of hyperandrogenemia, is connected to the severity of its health implications [1]. Apart from the well-known reproductive manifestations, PCOS has been associated with an increased risk of metabolic disorders, including insulin resistance, central obesity, and cardiovascular disease [2,3]. Additionally, several studies reported that PCOS patients have an increased risk of various psychiatric disorders, including, but not limited to, depression [4,5]. Taking into consideration the diversity in PCOS phenotypes, there are differences in its treatment, and therapy should be individualized [1].

Beyond the classical metabolic disorders, women with PCOS also manifest a disruption in amino acid metabolism [6]. Amino acids (AAs) are molecules that are essential in polypeptide and protein synthesis. Additionally, they are involved in various physiological processes as regulating factors and play an important role in maintaining homeostasis [7]. Among amino acids, there are molecules which participate directly or through their metabolites in neurotransmission, the regulation of gene expression, or antioxidation [7]. On the other hand, it has also been suggested that an alteration in the amino acid profile might be a predictive sign of other metabolic disturbances [8,9]. “Functional AAs” is a term used for a group of amino acids which are involved in the regulation of metabolic pathways; leucine, tryptophan, glutamine, proline, cysteine, and arginine are included in this category [7]. Another classification of AAs is based on the chemical structure of the molecule, which includes branched-chain amino acids (BCAAs) and aromatic amino acids (AAAs), both analyzed in the present study. 

Increased serum BCAA concentrations were previously described in patients with obesity, insulin resistance, or type 2 diabetes [10], as well as, recently, in women with PCOS [5]. Interestingly, BCAA concentrations in obese individuals tend to decrease following weight loss due to both lifestyle modifications and gastric bypass surgery [11,12]. An elevated BCAA level was also associated with a susceptibility to IR irrespective of obesity in normoglycemic women [13]. An increase in BCAAs was additionally suspected to potentially promote IR through the disruption of insulin signaling in myocytes [10]. Simultaneously, significant changes in AAA level were found in PCOS women [14], as well as in patients diagnosed with metabolic syndrome [15]. Tryptophan is one of the AAAs, mainly metabolized in humans through the kynurenine pathway [16]. An overly activated tryptophan–kynurenine pathway was already shown to exist in cardiovascular disease, obesity, type 2 diabetes mellitus, and PCOS [17,18,19,20], and the up-regulation of enzymes from this pathway was found to be connected to metabolic disturbances and might play a physiological, protective role [19].

PCOS women have a significantly higher risk of becoming overweight and developing obesity when compared to age-matched non-PCOS individuals [21], and the body mass index (BMI) is a simple formula calculated based on an individual’s weight and height that allows the classification of overweight and obesity, according to WHO recommendations [22]. The BMI does not directly assess body fat mass, but there is evidence indicating a relationship between BMI level and adverse effects on health [23]. From the individual point of view, BMI is not a prognostic marker to evaluate potential comorbidities as it does not indicate heterogeneity in the distribution of body fat [24,25]; this limitation results in a notable number of people classified as overweight and obese who do not develop metabolic and cardiovascular complications. As a consequence, metabolically healthy obesity (MHO) and metabolically unhealthy obesity (MUO) are concepts that have attracted scientific attention; however, a universal definition has not been established yet [26] for these terms. Already existing definitions of MHO involve the coexistence of obesity with the absence or minimal presence of other metabolic syndrome parameters [25,26].

Individuals assessed as having MHO tend to present decreased levels of systemic inflammation markers, although their fat mass does not differ compared to people with MUO [27]. Moreover, there are studies describing a lower risk of developing type 2 diabetes mellitus or cardiovascular disease among MHO patients, but, on the other hand, it is still higher than in metabolically healthy, normal-weight people [28]. Assessment of MHO and MUO PCOS women revealed differences not only in classic metabolic parameters between those groups but also in novel markers, such as the Visceral Adiposity Index (VAI), and Fatty Liver Index (FLI) as well [29].

An alteration in the amino acid profile was suggested as having a potential role in distinguishing PCOS women from controls in both normal-weight and obese populations [30]; however, the underlying mechanism has not been elucidated yet.

In the present study, we evaluated the plasma concentrations of BCAAs and AAAs as potential biomarkers of early metabolic disorders connected to PCOS and assessed a relationship between the amino acid profile and metabolic disturbances.

## 2. Material and Methods

The methodology of this study is consistent with the one presented in the previous study [31]. 

### 2.1. Study and Control Groups

The study population of Caucasian ethnicity included 208 patients diagnosed with PCOS and 118 individuals in the control group. Inclusion criteria were age between 18 and 40 years old, no history of diabetic or hypolipemic therapy, and not taking hormonal contraception within 6 months before tests were performed. Women classified as control group had regular cycles, and no abnormalities in ovarian morphology assessed in ultrasound examination. The diagnosis of PCOS was made based on the revised 2003 Rotterdam criteria [32]. The Bioethics Committee of the Medical University of Gdańsk (permission number NKBBN/27/2018) approved the study, and all women provided written consent.

### 2.2. Anthropometric Parameters

Weight, height, and waist circumference were assessed with standard techniques. BMI was calculated with a formula in which patient weight in kilograms was divided by height in meters squared. Obesity was assessed as BMI of 30 or above, according to the WHO classification [22]. In the study population, 67 women were diagnosed with obesity; in this group, there were 46 PCOS and 21 control individuals.

Abdominal obesity was diagnosed based on waist circumference (WC) greater or equal to 80 cm. Subsequently, a group of women with abdominal obesity (AbO+) was separated and included 143 PCOS and 74 control individuals.

MUO was defined based on the National Cholesterol Education Program Adult Treatment Panel III (NCEP ATP III) definition of metabolic syndrome as the presence of at least two out of the four diagnostic criteria, excluding waist circumference [33], and 19 PCOS individuals were included in this group; 27 women with PCOS who met fewer than two criteria were classified as MHO. 

### 2.3. Biochemical and Hormonal Assessment

Blood samples were collected after overnight fasting; biochemical and hormonal measurements were performed using commercial methods. 

Homeostatic model assessment of insulin resistance (HOMA IR) was used to assess insulin resistance and IR was defined as HOMA IR above 2.5 [34,35,36], and, among the study population, 115 women met this criterion, including 85 PCOS patients and 30 controls.

Lipid accumulation product (LAP) was calculated based on following: LAP = (waist circumference [cm] − 58) × (triglyceride concentration [mmol/L]);

Atherogenic Index of Plasma (AIP) was calculated with the formula: AIP = lg(TG [mmol/L]/HDL [mmol/L])

Free androgen index (FAI) was assessed based on the formula FAI = total testosterone [nmol/L] × 100/SHBG [nmol/L], and reference range 0.6–4.4 was applied [37]. Hyperandrogenemia was defined as a concentration of total testosterone and/or androstenedione and/or FAI above the reference range. 

PCOS patients were evaluated for hyperandrogenemia using the criteria mentioned above and divided into subgroups with hyperandrogenemia (HA+), including 121 women, and without hyperandrogenemia (HA−), including 87 individuals.

### 2.4. Branched-Chain and Aromatic Amino Acid Profile Assessment

BCAA and AAA concentrations were assessed using gas chromatography combined with mass spectrometry (GC/MS). Commercially available EZ:faast amino acid analysis kit (Phenomenex) was used for amino acid concentration determination according to manufacturer’s manual as published previously [38]. Briefly, serum samples after addition of norvaline as internal standard were subjected to solid phase extraction, washing with n-propanol, and eluting with a mixture of NaOH, n-propanol, and 3-picoline, followed by a derivatization of amino acids with propyl chloroformate. After the reaction and liquid/liquid extraction using isooctane, samples were analyzed using GC-MS system from ThermoFisher (ThermoFisher Scientific, Austin, TX, USA): focus gas chromatograph equipped with the column ZB-AAA GC (10 m, 0.25 mm ID, Phenomenex) and connected to mass spectrometer ITQ 700. For quantitation purposes, three level calibration standard curves were used and calculations were based on internal standard method (internal standard: norvaline). Peak identifications were carried out by retention time and MS spectra matching to Calibration Solution Mix supplied with EZ:faast Kit. All measurements (samples and standards) were performed in triplicate.

Tryptophan ratio is defined as the concentration of tryptophan divided by the sum of valine, leucine, isoleucine, phenylalanine, and tyrosine levels.

### 2.5. Statistical Analysis

Statistica (TIBCO), version 13.3, was used for data analysis. Differences between the groups were calculated using *t*-test and the Mann–Whitney U test for normally and non-normally dispersed parameters, respectively. The Spearman correlation was used to assess correlation between parameters. In all analyses, *p* < 0.05 was considered statistically significant.

## 3. Results

The baseline information (anthropometric, hormonal, and biochemical parameters) is presented in Table 1 as previously published [3]. Compared with healthy individuals, several parameters were significantly higher in PCOS patients, including LH, prolactin, testosterone, DHEA-S, FAI, androstenedione, HOMA-IR, and albumin. On the other hand, anthropometric and metabolic parameters, excluding age and HOMA-IR, do not differ noticeably between study groups. 

Current statistical analysis revealed noticeably higher levels of all aromatic amino acids in PCOS women compared to the control group: phenylalanine 47.37 ± 7.0 vs. 45.4 ± 6.09 nmol/mL (*p* = 0.01), tyrosine 61.69 ± 9.56 vs. 58.08 ± 8.89 nmol/mL (*p* < 0.01), and tryptophan 53.66 ± 11.42 vs. 49.81 ± 11.18 nmol/mL (*p* < 0.01). 

Table 2 presents the differences in AA concentrations between PCOS and healthy individuals in the study subpopulations: women with insulin resistance, abdominal obesity, or obesity. It was found that, in all listed subpopulations, the tryptophan concentration and level of aromatic amino acids analyzed as a group were significantly increased.

In the study subpopulation of women without insulin resistance, it was found that only tryptophan and isoleucine concentrations differ significantly between PCOS and healthy individuals, with *p* = 0.04 and *p* = 0.02, respectively.

The amino acid profile was assessed in the PCOS group between women with (IR+) and without insulin resistance (IR-). All analyzed amino acids, except for tryptophan, had significantly higher concentrations in the IR+ group. Results, including HOMA-IR and FAI, are presented in Table 3.

As shown in Table 4, similar results were observed in the PCOS group between patients with and without obesity. It is worth noting that obese women had significantly higher HOMA-IR (3.69 ± 1.75 vs. 2.22 ± 1.28; *p* < 0.001) and FAI (6.72 ± 4.71 vs. 3.21 ± 2.19; *p* < 0.001).

There were no significant differences in the “tryptophan ratio” between the PCOS and control group (*p* = 0.88); however, in the study population, the tryptophan ratio was significantly lower in obese individuals when compared to non-obese subjects (0.075 ± 0.018 vs. 0.086 ± 0.018; *p* < 0.001), and in women with insulin resistance compared to those without IR (0.078 ± 0.019 vs. 0.087 ± 0.018; *p* < 0.001). A positive correlation was found between the tryptophan ratio and percentage of body muscle mass (S = 0.38; *p* < 0.05), and a negative correlation with the percentage of body fat mass (S = −0.39; *p* < 0.05), BMI (S = −0.38; *p* < 0.05), HOMA-IR (S = −0.28; *p* < 0.05), and FAI (S = −0.21; *p* < 0.05).

The next step in the statistical analysis was to assess differences in the amino acid profile between metabolically healthy obesity (MHO) and metabolically unhealthy obesity (MUO) PCOS patients. As presented in Table 5, it was found that MUO PCOS women had higher concentrations of leucine and isoleucine; there were no significant differences in the levels of other AAs. 

An analysis of the correlation between the biochemical and anthropometrical parameters and amino acid profile showed significant relationships between AIP, BMI, LAP, FAI, and HOMA-IR, and both BCAA and AAA. The Spearman correlation coefficient is presented in Table 6.

The AAA levels were subsequently analyzed in the PCOS population between women with (HA+) and without (HA−) hyperandrogenemia, and no significant differences were found. The results are presented in Table 7. 

## 4. Discussion

In the current study, several alterations in the amino acid profile have been found in patients diagnosed with PCOS; concentrations of aromatic amino acids, analyzed as a group or separately, were increased in PCOS women compared to healthy individuals. Moreover, in the subpopulations of women with obesity (Ob+), abdominal obesity (AbO+), or insulin resistance (IR+), there was observed a tendency towards an increased level of all aromatic amino acids in individuals with PCOS; however, only an increase in tryptophan concentration was statistically significant. 

The results are in agreement with the previous research [30,39,40,41], in which higher AAA serum concentrations were reported in the PCOS population. However, the partial differences appear with regard to the AAA level in women with obesity. It was reported that, among obese women diagnosed with metabolic syndrome, tyrosine level was associated with PCOS and the sum of phenylalanine and tyrosine was higher in PCOS patients [42], whereas, in our study, the differences in tryptophan level were the most significant. A lack of conformity in the results might be caused by different inclusion criteria and the severity of metabolic disturbances in the study groups; in our study, not all obese patients were diagnosed with metabolic syndrome.

Among our PCOS group, individuals with insulin resistance had significantly higher concentrations of all BCAAs, phenylalanine, and tyrosine; however, tryptophan level does not vary between the groups. Similar results were observed in the subgroup of obese PCOS patients when compared to non-obese ones. Subsequently, an analysis of the AA profile of obese PCOS women revealed that patients presenting with MUO had significantly higher leucine and isoleucine levels and no differences in the concentrations of other analyzed amino acids. These results suggest an important correlation between metabolic health and average plasma levels of leucine and isoleucine. The underlying mechanisms behind the changes in serum BCAA concentrations among the obese population is not well understood but it is suggested that it might be connected to the altered metabolism of BCAAs in adipocytes of the white adipose tissue [43]. Leucine accounts for approximately 10% of tissue protein and its plasma level may increase rapidly during catabolic states [44]; its concentration was significantly associated with muscle mass and strength and also negatively correlated with sarcopenic risk [45]. The severe accumulation of all BCAAs due to branched-chain α-ketoacid dehydrogenase (BCKD) deficiency is a cause of maple syrup urine disease (MSUD). The clinical picture of this rare genetic disorder includes neuropsychiatric disorders, such as ataxia, as well as mental and psychomotor retardation, liver insufficiency, and altered carbohydrates metabolism [46], which is a biological presentation of the toxic effect of massive BCAA excess. A mild increase in the BCAA level found in PCOS is connected with much less intense adverse effects; however, a negative impact on metabolic health is observed. While analyzing the disturbances in BCAA metabolism, individuals with PCOS and those diagnosed with MSUD represent opposite extremes of the continuum of BCAA accumulation in the human body.

A significant positive correlation of BCAA and AAA levels with surrogate markers of visceral adiposity (LAP) and plasma atherogenic activity (AIP) was found, and, as far as we know, these correlations have not been reported previously. AIP was introduced as one of the biomarkers to predict cardiovascular disease as it is negatively correlated with the size of the lipoprotein particle [47]. Moreover, a relationship between AIP and metabolic disorders, such as obesity, diabetes, and metabolic syndrome, was reported [48,49,50]. The LAP index represents impaired fat distribution, and a value equal or higher than 34.5 was suggested as indicative of a possible risk factor for cardiovascular disease in PCOS [51]. The associations between both BCAAs and AAAs with the mentioned surrogate markers of cardiometabolic issues requires further investigations.

Hyperandrogenemia is a part of the clinical picture of PCOS and has a great impact on the metabolism. Sexual dimorphism is observed in the testosterone influence on metabolic processes. On the one hand, a low concentration of testosterone is connected to hyperinsulinemia and insulin resistance in men, and testosterone replacement therapy improves the insulin sensitivity in hypogonadal men [52,53]; on the other hand, in women, androgen excess is correlated with insulin resistance, and free testosterone level in adolescent girls was suggested as a risk factor of metabolic syndrome development [54]. 

In the present study, concentrations of aromatic amino acids in the PCOS group do not differ significantly between women with and without hyperandrogenemia, but, on the other hand, a weak positive correlation between AAA level and total testosterone, as well as FAI, was found. In the literature, levels of all tryptophan–kynurenine metabolites in urine or plasma measurements of PCOS women were positively correlated with FAI and plasma testosterone level [20,55].

A significantly increased concentration of BCAAs was previously reported in PCOS [30,39,40,41], and our data are consistent [31]. The chronic elevation of BCAA levels was suspected to impair the transport of aromatic amino acids into cells and, as a result, to reduce the production of particular neurotransmitters [10]. That may also lead to an imbalance between Trp level and its neutral amino acids competitors (Phe, Leu, Ile, Val, and Tyr); a decreased “tryptophan ratio” was observed in obesity [10,56,57] and depression [58,59].

Although, in the present study, the median tryptophan level was significantly higher in PCOS patients, there was no difference in the tryptophan ratio between PCOS and healthy women. On the other hand, in the study population, there was a significant reduction in the tryptophan ratio in obese when compared to non-obese individuals and in insulin-resistant women when compared to those with a normal insulin sensitivity, which is consistent with the literature data [10,57]. The increase in tryptophan level was previously reported in PCOS women in plasma [20] and ovarian follicular fluid [60], but, as far as we know, the tryptophan ratio has not been assessed previously in the PCOS population and it was first counted in PCOS in the present study. 

The increased tryptophan concentration and, surprisingly, no change in the tryptophan ratio in PCOS women might suggest a mechanism for developing this disturbance independent of insulin resistance and in a manner not related to the BCAA and AAA transport system, which is important in regulating neurotransmitter biosynthesis. This finding supports the hypothesis that metabolic disturbances observed in PCOS have a complex etiology and not all of them simply arise from insulin resistance and obesity; additionally, it indicates a possibility that an impaired tryptophan metabolism might be involved in PCOS pathogenesis. This observation is consistent with the literature data that tryptophan metabolism is affected in PCOS regardless of body mass [20].

Tryptophan metabolism involves two pathways: one for serotonin and, subsequently, melatonin synthesis; and the kynurenine pathway, which is responsible for the catabolism of approximately 95% of tryptophan [61]. The kynurenine pathway includes intermediate metabolites, such as kynurenine, 3-hydroxykynurenine, 3-hydroxyanthranilic acid, quinolinic acid, or kynurenic acid [62]. It was reported that hypertension, obesity, and diabetes are associated with higher levels of metabolites from the tryptophan–kynurenine pathway [63]. Additionally, there is evidence suggesting that tryptophan metabolism might be dysregulated in PCOS. In the present study, tryptophan catabolites were not assessed but metabolomic research showed disturbances in the kynurenine pathway in PCOS manifested by the up-regulation of tryptophan and its catabolites [20,47]. Moreover, modifications in the enzymatic activity of indoleamine 2,3-dioxygenase (IDO), the rate-limiting enzyme of the kynurenine pathway, were reported in PCOS, which may lead to important imbalances between metabolites of the two pathways engaged in tryptophan metabolism [20].

The prevalence of depressive and anxiety symptoms is approximately 14 to 67% among PCOS women, whereas, in the age-matched general population, it is typically 4–6% [64,65]. It was also found that the prevalence of depression remains significantly higher during the life-time in PCOS patients [66]. Although the psychosocial aspects of a PCOS diagnosis are involved in the development of depressive symptoms, the dysregulation of tryptophan metabolism may be potentially connected with the higher incidence of depression among PCOS individuals. First of all, the increased concentration of metabolites in the kynurenine pathway seems to be associated with a neurotoxic effect in CNS; it was found that quinolinic acid induces the production of reactive oxygen species and pro-inflammatory cytokines [67]. Additionally, the alteration in tryptophan metabolism may be connected with an imbalance in serotonin synthesis in the central nervous system and, as a consequence, affects mood regulation.

## 5. Limitations

The present study has several limitations. First of all, the subgroups of PCOS women with MHO and MUO included a small number of subjects, and that may affect the statistical calculations. Moreover, only amino acid concentrations were measured, without an assessment of their metabolites, which could have an additional impact. Finally, it still remains unclear whether the reported alteration of the AA profile is a consequence of other metabolic and hormonal disturbances, or if it is a part of PCOS pathogenesis.

## 6. Conclusions

The identification of altered amino acid profiles in PCOS holds potential clinical implications. Amino acids may serve as biomarkers for diagnosing and monitoring the metabolic status of individuals with PCOS. Furthermore, targeting amino acid metabolism could provide new therapeutic approaches for the management of PCOS and associated metabolic complications. The alteration of BCAAs and AAAs may be involved in PCOS pathogenesis but the underlying mechanism should be further explored. An investigation of the enzymes involved in the regulation of amino acid metabolism is one of the promising approaches in future studies.

## Figures and Tables

**Table 1:** Anthropometric, biochemical, and hormonal data of the study subjects.

	PCOS	Control	*p*
age	25.86 ± 5.38	31.08 ± 6.99	<0.001
BMI [kg/m^2^]	26.09 ± 6.37	25.39 ± 5.22	0.67
waist circumference [cm]	89.75 ± 15.16	87.17 ± 14.59	0.12
fasting glucose [mg/dL]	87.22 ± 6.56	86.97 ± 8.37	0.41
HDL [mg/dL]	65.69 ± 18.44	66.97 ± 15.64	0.40
triglycerides [mg/dL]	95.39 ± 60.55	86.79 ± 42.61	0.31
total cholesterol [mg/dL]	188.23 ± 35.60	188.89 ± 35.71	0.86
LDL [mg/dL]	104.47 ± 33.37	104.46 ± 32.52	0.81
albumin [mg/mL]	48.09 ± 2.85	47.35 ± 2.62	0.03
non-HDL [mg/dL]	122.65 ± 37.94	121.91 ± 35.18	0.83
CRP [mg/L]	1.78 ± 3.28	1.72 ± 2.54	0.88
WBC	6.26 ± 1.57	5.67 ± 1.35	0.001
TSH [mU/L]	2.49 ± 1.55	2.10 ± 1.30	0.02
LH [mIU/mL]	9.57 ± 7.40	7.09 ± 6.03	<0.001
FSH [mIU/mL]	6.85 ± 4.05	6.76 ± 2.30	0.85
LH/FSH	1.46 ± 1.04	1.13 ± 0.98	<0.001
estradiol [pg/mL]	232.79 ± 151.47	391.13 ± 179.84	<0.002
prolactin [uIU/L]	433.11 ± 194.11	372.35 ± 152.38	0.006
DHEA-S [ug/dL]	314.53 ± 125.92	204.22 ± 74.25	<0.001
testosterone [nmol/L]	1.86 ± 0.67	1.05 ± 0.33	<0.001
SHBG [nmol/L]	65.75 ± 38.41	76.77 ± 37.17	0.001
fasting insulin [mU/mL]	11.83 ± 6.89	8.91 ± 4.80	<0.001
FAI	3.99 ± 3.29	1.75 ± 1.28	<0.001
androstenedione [ng/mL]	3.28 ± 1.31	2.13 ± 0.82	<0.001
HOMA-IR	2.55 ± 1.53	1.96 ± 1.15	<0.001
LAP	38.13 ± 35.39	29.35 ± 21.85	0.14
AIP	0.14 ± 0.28	0.1 ± 0.2	0.36

BMI—Body Mass Index, CRP—C-reactive protein, WBC—white blood cells, TSH—thyroid stimulating hormone, LH—luteinizing hormone, FSH—follicle-stimulating hormone, DHEA-S—dehydroepiandrosterone sulfate, SHBG—sex-hormone-binding globulin, FAI—free androgen index, HOMA-IR—homeostatic model assessment of insulin resistance, LAP—lipid accumulation product, AIP—Atherogenic Index of Plasma. In our study, PCOS women had significantly higher BCAA concentration in comparison to the control group. The differences between study groups were still observed in the subpopulations of patients with insulin resistance or abdominal obesity but not among obese women.

**Table 2:** Comparison of AAA level between PCOS and control groups in the subpopulations of women with insulin resistance, abdominal obesity, and obesity.

Women with Insulin Resistance (IR+)			
	PCOS	Control	*p*
Phenylalanine [nmol/mL]	48.36 ± 6.72	45.86 ± 6.27	0.08
Tyrosine [nmol/mL]	65.97 ± 9.65	60.17 ± 8.73	<0.01
Tryptophan [nmol/mL]	53.62 ± 11.12	48.82 ± 15.0	0.03
AAA [nmol/mL]	167.95 ± 22.64	154.85 ± 24.17	<0.01
Women with abdominal obesity (AbO+)			
Phenylalanine [nmol/mL]	47.52 ± 6.41	46.33 ± 5.79	0.15
Tyrosine [nmol/mL]	62.56 ± 9.90	60.78 ± 8.57	0.20
Tryptophan [nmol/mL]	53.03 ± 10.40	49.55 ± 12.34	<0.01
AAA [nmol/mL]	163.11 ± 21.41	156.66 ± 20.13	0.01
Women with obesity (Ob+)			
Phenylalanine [nmol/mL]	48.79 ± 6.97	48.14 ± 6.33	0.74
Tyrosine [nmol/mL]	67.57 ± 9.54	61.68 ± 7.84	0.02
Tryptophan [nmol/mL]	53.03 ± 9.69	48.63 ± 14.59	0.02
AAA [nmol/mL]	169.38 ± 21.57	158.44 ± 24.53	0.03

AAA—aromatic amino acid.

**Table 3:** Differences in AA concentrations, HOMA-IR, and FAI between women with and without insulin resistance among PCOS group.

	IR+ (86)	IR− (123)	*p*
BCAA [nmol/mL]	579.01 ± 102.32	513.47 ± 83.48	<0.001
Val [nmol/mL]	356.48 ± 66.91	313.14 ± 56.72	<0.001
Leu [nmol/mL]	139.19 ± 23.77	126.58 ± 19.29	<0.001
Ile [nmol/mL]	83.35 ± 17.69	72.84 ± 12.87	<0.001
AAA [nmol/mL]	168.04 ± 22.53	159.10 ± 21.98	<0.001
Phe [nmol/mL]	48.41 ± 6.70	46.68 ± 7.14	0.01
Tyr [nmol/mL]	65.94 ± 9.60	58.74 ± 8.32	<0.001
Trp [nmol/mL]	53.70 ± 11.08	53.68 ± 11.66	0.80
HOMA-IR	3.94 ± 1.36	1.56 ± 0.56	<0.001
FAI	5.63 ± 4.06	2.83 ± 1.85	<0.001

BCAA—branched-chain amino acid, Val—valine, Leu—leucine, Ile—isoleucine, AAA—aromatic amino acid, Phe—phenylalanine, Tyr—tyrosine, Trp—tryptophan, HOMA-IR—homeostatic model assessment of insulin resistance, FAI—free androgen index.

**Table 4:** Differences in AA concentrations between obese and non-obese women among PCOS group.

	PCOS Ob+ (46)	PCOS Ob− (163)	*p*
BCAA [nmol/mL]	596.31 ± 109.08	524.68 ± 87.43	<0.001
Val [nmol/mL]	365.59 ± 71.27	321.21 ± 59.26	<0.001
Leu [nmol/mL]	143.86 ± 25.99	128.36 ± 19.63	<0.001
Ile [nmol/mL]	86.86 ± 17.91	75.11 ± 14.08	<0.001
AAA [nmol/mL]	169.38 ± 21.57	160.92 ± 22.58	0.007
Phe [nmol/mL]	48.79 ± 6.97	47.0 ± 6.97	0.03
Tyr [nmol/mL]	67.57 ± 9.54	60.04 ± 8.88	<0.001
Trp [nmol/mL]	53.03 ± 9.69	53.88 ± 11.85	0.53

BCAA—branched-chain amino acid, Val—valine, Leu—leucine, Ile—isoleucine, AAA—aromatic amino acid, Phe—phenylalanine, Tyr—tyrosine, Trp—tryptophan.

**Table 5:** Comparison of amino acid concentrations between PCOS patients with metabolically healthy obesity (MHO) and metabolically unhealthy obesity (MUO).

	MHO (27)	MUO (19)	*p*
BCAA [nmol/mL]	578.41 ± 114.60	621.75 ± 98.03	0.15
Val [nmol/mL]	358.56 ± 74.26	375.58 ± 67.47	0.45
Leu [nmol/mL]	137.25 ± 26.76	153.26 ± 22.26	0.04
Ile [nmol/mL]	82.60 ± 18.17	92.92 ± 16.09	0.02
AAA [nmol/mL]	166.92 ± 16.82	172.89 ± 27.06	0.57
Phe [nmol/mL]	47.29 ± 5.63	50.90 ± 8.23	0.12
Tyr [nmol/mL]	67.30 ± 8.22	67.94 ± 11.38	0.95
Trp [nmol/mL]	52.32 ± 8.23	54.04 ± 11.63	0.88

BCAA—branched-chain amino acid, Val—valine, Leu—leucine, Ile—isoleucine, AAA—aromatic amino acid, Phe—phenylalanine, Tyr—tyrosine, Trp—tryptophan.

**Table 6:** Correlation of biochemical, hormonal, and anthropometrical parameters with BCAA and AAA plasma concentrations presented as Spearman correlation coefficient.

	BCAA	AAA	*p*
AIP	0.28	0.16	<0.05
BMI	0.32	0.14	<0.05
LAP	0.39	0.24	<0.05
FAI	0.34	0.18	<0.05
HOMA-IR	0.36	0.21	<0.05

AIP—Atherogenic Index of Plasma, BMI—Body Mass Index, LAP—lipid accumulation product, FAI—free androgen index, HOMA-IR—homeostatic model assessment of insulin resistance.

**Table 7:** Comparison of aromatic amino acid concentrations in PCOS population between women with (HA+) and without hyperandrogenemia (HA−).

	PCOS HA+ (122)	PCOS HA− (87)	*p*
AAA [nmol/mL]	162.80 ± 21.63	162.75 ± 24.0	0.62
Phe [nmol/mL]	47.65 ± 6.81	47.04 ± 7.28	0.20
Tyr [nmol/mL]	61.64 ± 9.90	61.79 ± 9.05	0.73
Trp [nmol/mL]	53.52 ± 10.43	53.93 ± 12.69	0.87

BCAA—branched-chain amino acid, Val—valine, Leu—leucine, Ile—isoleucine, AAA—aromatic amino acid, Phe—phenylalanine, Tyr—tyrosine, Trp—tryptophan.

## Data Availability

The data presented in this study are available on request from the corresponding author. The data are not publicly available due to privacy and ethical reasons.
